# Case of Carimoma of the Pancreas and Omentum in an Oclos-Feli-Pardalis

**Published:** 1880-10

**Authors:** 


A Case of Carimoma of the Pancreas and Omentum in an Oclos-Feli-Pardalis of South America—[Central Park ZoölogicalGarden.]

The following brief history of the case was obtained from the Superintendent, Mr. William C. Conklin. The animal had been in the Garden only a few months. It was only a week or two prior to death that the animal showed any symptoms of disease. The first thing noticed, was loss of appetite, accompanied by vomiting. Languor and loss of strength followed. Oleum Recini was administered, which seemed to alleviate the symptoms and produce temporary improvement. All treatment, however, failed to secure any permanent good; the animal rapidly sank and died, June 12th, 1880. A necropsy was held on the animal by Dr. E. C. Wendt, in the presence of Superintendent Conklin and Dr. Porter.

*Necropsy* thirteen hours after death.—Rigor mortis well marked; body very much emaciated; the contents of the thoracic cavity appeared normal. *Abdominal Cavity.* —When the abdomen was opened considerable congestion and thickening of the peritoneum covering the abdominal surface of the diaphragm was found. Upon the surface and in the substance of the pancreas there were a number of hard white masses, evidently new growths. The omentum and mesentery in the immediate vicinity of the pancreas was studded with similar white masses. There was a tumor the size of a walnut lying close to the intestine one inch below the pylorous. It was the opinion of those present that the new growth was of a cancerous nature; that death of the animal was due to exhaustion.—[Notes by W. H. P.]

